# Characterization of Influenza Virus Pseudotyped with Ebolavirus Glycoprotein

**DOI:** 10.1128/JVI.00941-17

**Published:** 2018-01-30

**Authors:** Julie Huiyuan Xiao, Pramila Rijal, Lisa Schimanski, Arun Kumar Tharkeshwar, Edward Wright, Wim Annaert, Alain Townsend

**Affiliations:** a Human Immunology Unit, Weatherall Institute of Molecular Medicine, Oxford, United Kingdom; b VIB-Center for Brain and Disease Research, Laboratory for Membrane Trafficking, Leuven, Belgium & KU Leuven, Department of Neurosciences, Leuven, Belgium; c Viral Pseudotype Unit, Faculty of Science and Technology, University of Westminster, London, United Kingdom; Icahn School of Medicine at Mount Sinai

**Keywords:** antiviral mechanism, Ebola virus, influenza pseudotype

## Abstract

We have produced a new Ebola virus pseudotype, E-S-FLU, that can be handled in biosafety level 1/2 containment for laboratory analysis. The E-S-FLU virus is a single-cycle influenza virus coated with Ebolavirus glycoprotein, and it encodes enhanced green fluorescence protein as a reporter that replaces the influenza virus hemagglutinin. MDCK-SIAT1 cells were transduced to express Ebolavirus glycoprotein as a stable transmembrane protein for E-S-FLU virus production. Infection of cells with the E-S-FLU virus was dependent on the Niemann-Pick C1 protein, which is the well-characterized receptor for Ebola virus entry at the late endosome/lysosome membrane. The E-S-FLU virus was neutralized specifically by an anti-Ebolavirus glycoprotein antibody and a variety of small drug molecules that are known to inhibit the entry of wild-type Ebola virus. To demonstrate the application of this new Ebola virus pseudotype, we show that a single laboratory batch was sufficient to screen a library (LOPAC^1280^; Sigma) of 1,280 pharmacologically active compounds for inhibition of virus entry. A total of 215 compounds inhibited E-S-FLU virus infection, while only 22 inhibited the control H5-S-FLU virus coated in H5 hemagglutinin. These inhibitory compounds have very dispersed targets and mechanisms of action, e.g., calcium channel blockers, estrogen receptor antagonists, antihistamines, serotonin uptake inhibitors, etc., and this correlates with inhibitor screening results obtained with other pseudotypes or wild-type Ebola virus in the literature. The E-S-FLU virus is a new tool for Ebola virus cell entry studies and is easily applied to high-throughput screening assays for small-molecule inhibitors or antibodies.

**IMPORTANCE** Ebola virus is in the Filoviridae family and is a biosafety level 4 pathogen. There are no FDA-approved therapeutics for Ebola virus. These characteristics warrant the development of surrogates for Ebola virus that can be handled in more convenient laboratory containment to study the biology of the virus and screen for inhibitors. Here we characterized a new surrogate, named E-S-FLU virus, that is based on a disabled influenza virus core coated with the Ebola virus surface protein but does not contain any genetic information from the Ebola virus itself. We show that E-S-FLU virus uses the same cell entry pathway as wild-type Ebola virus. As an example of the ease of use of E-S-FLU virus in biosafety level 1/2 containment, we showed that a single production batch could provide enough surrogate virus to screen a standard small-molecule library of 1,280 candidates for inhibitors of viral entry.

## INTRODUCTION

Ebola virus is a filamentous RNA virus that belongs to the Filoviridae family (1). It has a negative-stranded RNA genome (19 kb) that contains seven genes. Ebola virus is a zoonotic virus, and the mechanism by which it is maintained in its natural reservoirs, such as fruit bats, is not fully understood (2). The first Ebola outbreak in a human population happened in Congo and Sudan in 1976. During that Ebola outbreak, *Zaire ebolavirus* was first isolated and characterized (3, 4). Since then, five species of ebolavirus have been identified: *Zaire*, *Sudan*, *Taï* *Forest*, *Bundibugyo*, and *Reston ebolavirus* (5). Ebola virus is highly infectious in human and nonhuman primates and causes a hemorrhagic fever with a fatality rate of 25 to 90% (1). The recent epidemic in 2014 and 2015 caused nearly 30,000 human infections and more than 11,000 deaths in West Africa (6). So far, there is no FDA-approved treatment or vaccine against Ebola virus disease, but the recombinant vesicular stomatitis virus glycoprotein (rVSV-GP) vaccine has shown very promising protection in the Guinea ring vaccination trial (7).

Although much attention has been drawn to Ebola virus research since then, direct handling of Ebola virus is limited to biosafety level 4 laboratories. Development of a safe substitute is very important and useful for high-throughput screening of therapeutics, diagnostic screening of neutralizing human sera, and understanding the entry mechanism of Ebola virus.

Ebola virus is a lipid-enveloped virus, and the Ebola virus glycoprotein (EBOV-GP) is the only protein present at the virus surface. EBOV-GP plays an important role in virus cell entry, and it is the key target for neutralization by antibodies (8). Currently available viral surrogates for EBOV, such as EBOV-GP-pseudotyped lentivirus (9) and VSV (10), expose EBOV-GP at the viral surface. However, EBOV-GP-pseudotyped viruses are still different from wild-type Ebola virus and vary in their biological properties and susceptibility to neutralizing antibodies. Recently, the National Institute of Biological Standards and Control has compared 22 different Ebola virus-based *in vitro* assays with the wild-type Ebola virus for neutralization by a panel of antibodies and sera. The results showed variable but generally poor correlations (11). Therefore, designing and comparing additional EBOV-GP-pseudotyped viruses are important to accurately determine the correlates of protection.

Here we describe a new Ebola virus pseudotype (E-S-FLU) based on a nonreplicating influenza virus, the S-FLU virus (12). Influenza virus is also a negative-strand RNA virus. The S-FLU virus has its hemagglutinin (HA) gene replaced with an enhanced green fluorescence protein (eGFP) reporter. We found that unlike other cell lines (13–20), MDCK-SIAT1 cells can stably express high levels of EBOV-GP without apparent toxicity. Pseudotyping is done by simply infecting MDCK-SIAT1 producer cell lines (21) that are stably transduced to express EBOV-GP with seed S-FLU virus. The expression of EBOV-GP in the producer cell line complements the defect in HA expression, and the S-FLU virus replicates to levels sufficient to perform drug inhibition or antibody neutralization assays without further concentration. The stable producer cell line allows easy production of the E-S-FLU virus without the need for repeated transfection for each round of virus production, in comparison to lentivirus pseudotyping and VSV pseudotyping methods. The E-S-FLU virus does not contain any genetic material from the Ebola virus and can infect cell lines that do not express EBOV-GP or influenza virus HA for only a single cycle. Therefore, it can be handled at biosafety level 1/2. We have shown that the E-S-FLU virus is easy to produce and that it resembles Ebola virus during the cell entry process. We also compared the E-S-FLU virus with recombinant wild-type Ebola virus in drug screening assays, and the results were highly correlated. The E-S-FLU virus is a useful surrogate for Ebola virus in *in vitro* studies.

## RESULTS

### Generation of EBOV-GP-pseudotyped S-FLU virus.

**FIG 1:**
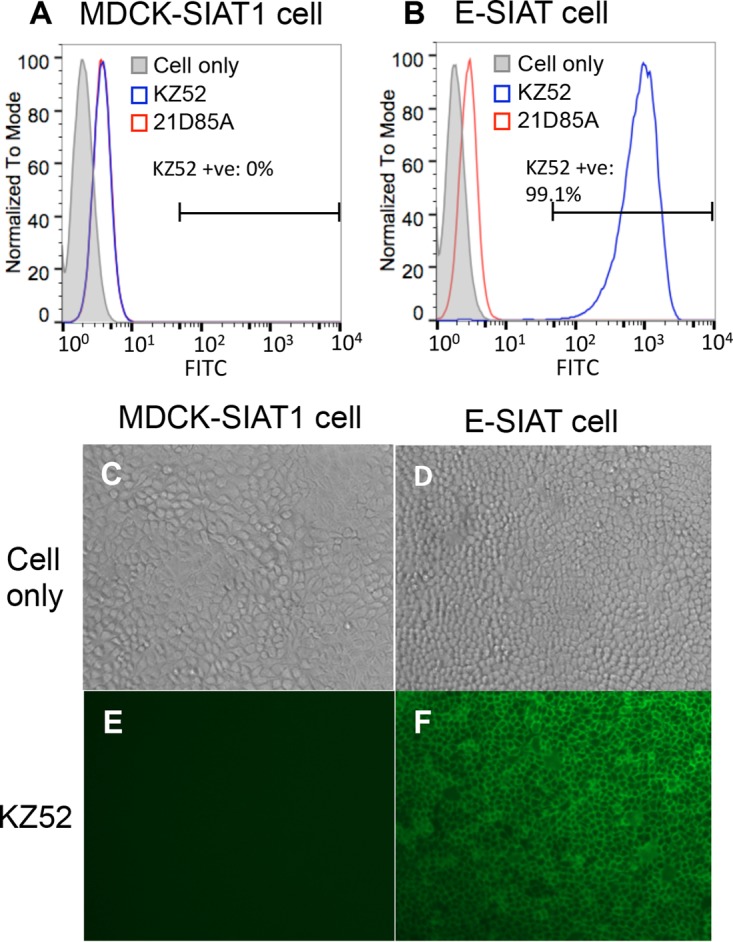
Stable expression of EBOV-GP on the surface of transduced MDCK-SIAT1 cells. Shown are FACS plots of MDCK-SIAT1 cells (A) and MDCK-SIAT1 cells transduced with full-length GP (E-SIAT) (B). Cells were stained with primary MAb KZ52 (anti-GP MAb) or 21D85A (anti-H5 MAb), followed by an FITC-linked anti-human secondary antibody. E-SIAT cells stably express GP at the cell surface up to at least the ninth passage, which can be detected with conformational MAb KZ52 specifically (B, blue). (C to F) Immunofluorescence pictures of MDCK-SIAT1 (C and E) and E-SIAT (D and F) cells stained with KZ52 and an FITC-linked anti-human antibody.

The S-FLU virus is a pseudotyped influenza virus with its endogenous HA sequence removed, and it was previously pseudotyped with different subtypes of influenza virus HA (12, 22). To incorporate EBOV-GP into the S-FLU virus envelope, we used a lentiviral vector to transduce MDCK-SIAT1 cells to express the full-length Zaire strain EBOV-GP gene (Zaire wt/GIN/2014/Kissidougou-C15 [Zaire C15]). Transduced cells were healthy in morphology (Fig. 1). EBOV-GP was expressed at the cell surface and can be detected with GP-specific monoclonal antibody (MAb) KZ52. Cells with high expression of EBOV-GP were sorted and stored as the E-SIAT cell line. GP surface expression was not toxic to MDCK-SIAT1 cells and was stable and uniform up to at least the ninth passage (Fig. 1).

**FIG 2:**
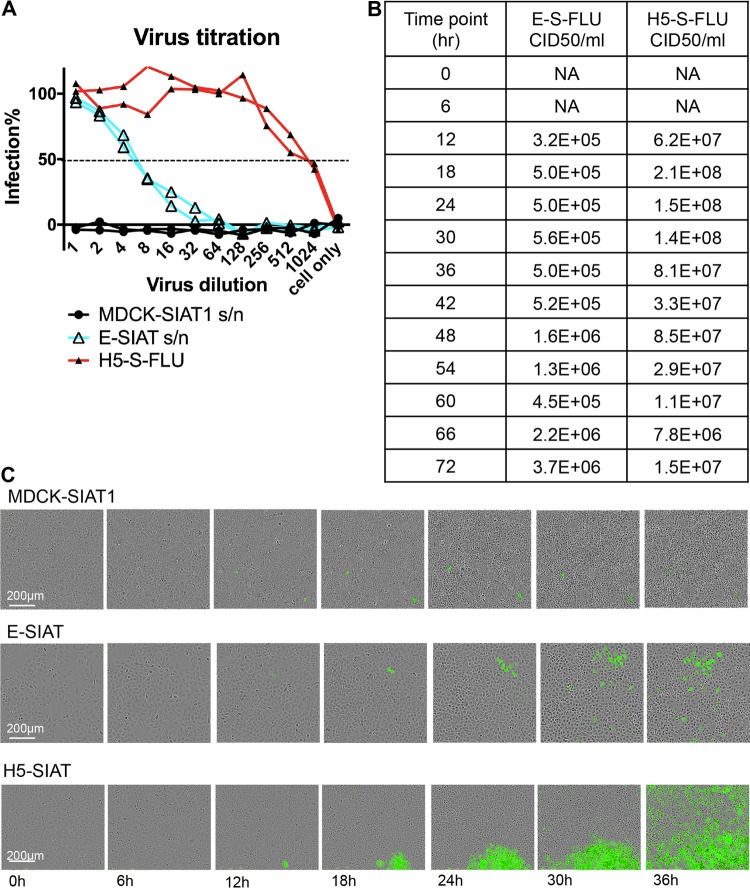
Pseudotyping of S-FLU virus with EBOV-GP. (A) E-SIAT cells were seeded with 1 TCID_50_ of seed S-FLU virus per cell to generate EBOV-GP (cyan)- and H5 (red)-pseudotyped S-FLU viruses. Parental MDCK-SIAT1 cells were also included as a control. Culture supernatant (s/n) harvested after 48 h was titrated in comparison to the H5-S-FLU virus in 2-fold dilutions on MDCK-SIAT1 indicator cells for 24 h of infection. Infected cells expressing eGFP were detected and quantified with a CLARIOstar fluorescence plate reader. The undiluted E-S-FLU and H5-S-FLU viruses gave saturated infection, and the maximum fluorescence readout was used to calculate the percent infection at lower dilutions. (B) E-SIAT and H5-SIAT cells were seeded with 1 TCID_50_ of seed S-FLU virus per cell. Culture supernatant harvested at different time points postseeding were titrated on MDCK-SIAT1 cells. The EC_50_ was calculated by linear interpolation. The CID_50_/ml was calculated from the EC_50_ dilution and the number of cells per well (3 × 10^4^). (C) A total of 100 TCID_50_ of seed S-FLU virus was added to 3 × 10^4^ MDCK-SIAT1, E-SIAT, or H5-SIAT cells. Live images of representative plaques were taken by IncuCyte every 3 h. E-SIAT cells support the formation of small diffuse plaques, unlike the dense plaques formed on H5-SIAT cells.

Cloned seed S-FLU virus that is coated in influenza virus HA was added to propagate in E-SIAT cells to produce the EBOV-GP-pseudotyped S-FLU virus. After 48 h, culture supernatants were harvested and titrated on indicator MDCK-SIAT1 cells for infection. Infected MDCK-SIAT1 cells express eGFP that is encoded in the virus. We named the new pseudotype E-S-FLU. The E-S-FLU virus reaches a lower titer than viruses grown in H5 HA-transduced producer cells and forms smaller, more diffuse plaques than the H5-S-FLU virus (Fig. 2C). The typical 50% effective concentration (EC_50_) dilutions of the E-S-FLU virus at 48 h were ∼1:8, compared to ∼1:500 for the H5-S-FLU virus (Fig. 2A). From the EC_50_ dilution and the number of cells per well (3 × 10^4^), the 50% cell infective dose (CID_50_)/ml was calculated as ∼2 × 10^6^ in the E-S-FLU virus batch and ∼1.6 × 10^8^ in the H5 S-FLU virus batch. However, the harvested culture supernatant from E-SIAT cells contains adequate pseudovirions for 50 μl of a 1:4 dilution to give close to full infection of the 3 × 10^4^ cells in a well of a 96-well plate. No further purification or concentration steps are needed for the screening of inhibitory drugs or antibodies. With the same protocol, we were also able to pseudotype the S-FLU virus with Bundibugyo, Sudan, and Mayinga-Zaire EBOV-GP to similar viral titers (data not shown).

It has been shown previously that the transmembrane and cytoplasmic domains of influenza virus HA are important for influenza viral particle assembly (23–30). To potentially improve the viral titer of the E-S-FLU virus, we designed a GP-HA hybrid protein that contains the extracellular domain of the EBOV-GP and the transmembrane and cytoplasmic domains of HA. After transduction and sorting, the GP-HA protein can be detected with MAb KZ52 at the surface of EH-SIAT cells at a level similar to that on E-SIAT cells. However, to our surprise, no infective S-FLU virus was detected in the EH-SIAT cell supernatant (data not shown).

### E-S-FLU virus infection requires NPC1 as a receptor.

**FIG 3:**
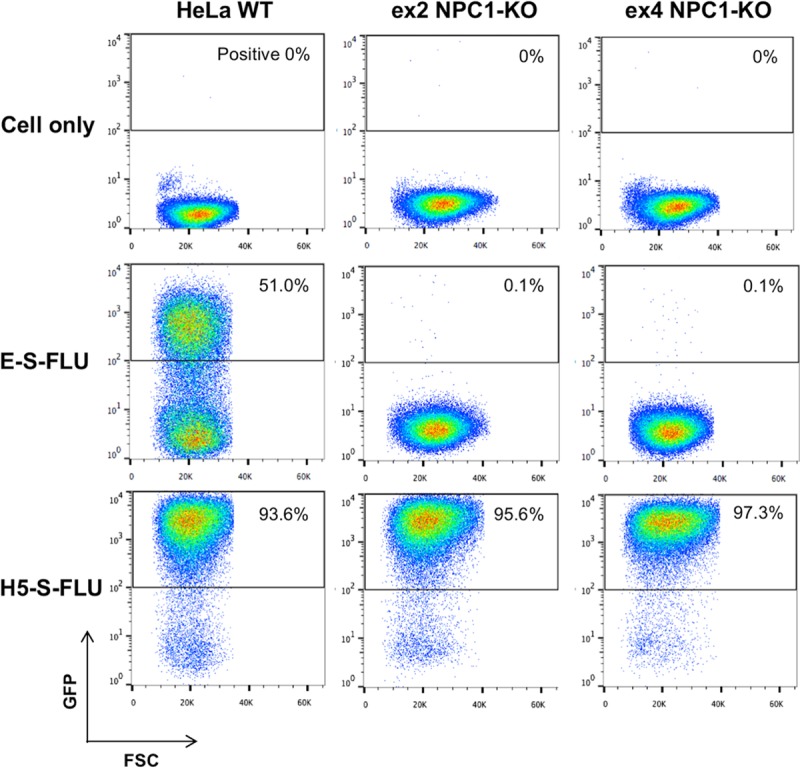
E-S-FLU virus requires NPC1 receptor expression for infection of HeLa cells. FACS plots of wild-type HeLa cells and two clones of NPC1 KO HeLa cells (ex2 NPC1-KO and ex4 NPC1-KO) infected with undiluted E-S-FLU and H5-S-FLU viruses. The S-FLU virus contains an eGFP reporter gene. The percentage of infected cells is the percentage of cells with a GFP readout of >10^2^. WT, wild type.

The Niemann Pick C1 (NPC1) protein has been identified as the key entry receptor for Ebola virus at the level of late endosome/lysosome membrane (31–33). We infected both wild-type HeLa cells and two NPC1 knockout (KO) HeLa cell lines (ex2 NPC1-KO and ex4 NPC1-KO) (34) with the E-S-FLU and H5-S-FLU viruses (Fig. 3). Wild-type HeLa cells were infected with the E-S-FLU virus and expressed eGFP. E-S-FLU virus infection of NPC1-KO cells was completely blocked. In contrast, the percent infection with the H5-S-FLU virus was the same for wild-type and NPC1-KO cells. This shows that the E-S-FLU virus requires NPC1 for cell entry, which resembles the wild-type Ebola virus cell entry mechanism.

### E-S-FLU virus is specifically neutralized by EBOV-GP antibody.

To further validate our new EBOV-GP-pseudotyped E-S-FLU virus, we tested it in the antibody microneutralization (MN) assay in comparison with control pseudotype H5-S-FLU virus and an EBOV-GP-coated lentivirus pseudotype (GP-lenti). The H5-S-FLU virus contains exactly the same S-FLU virus core as the E-S-FLU virus but differs at the surface glycoprotein. GP-lenti is a lentivirus pseudotyped with the full-length Zaire strain EBOV-GP (wt/GIN/2014/Kissidougou-C15) matched to the E-S-FLU virus.

**FIG 4:**
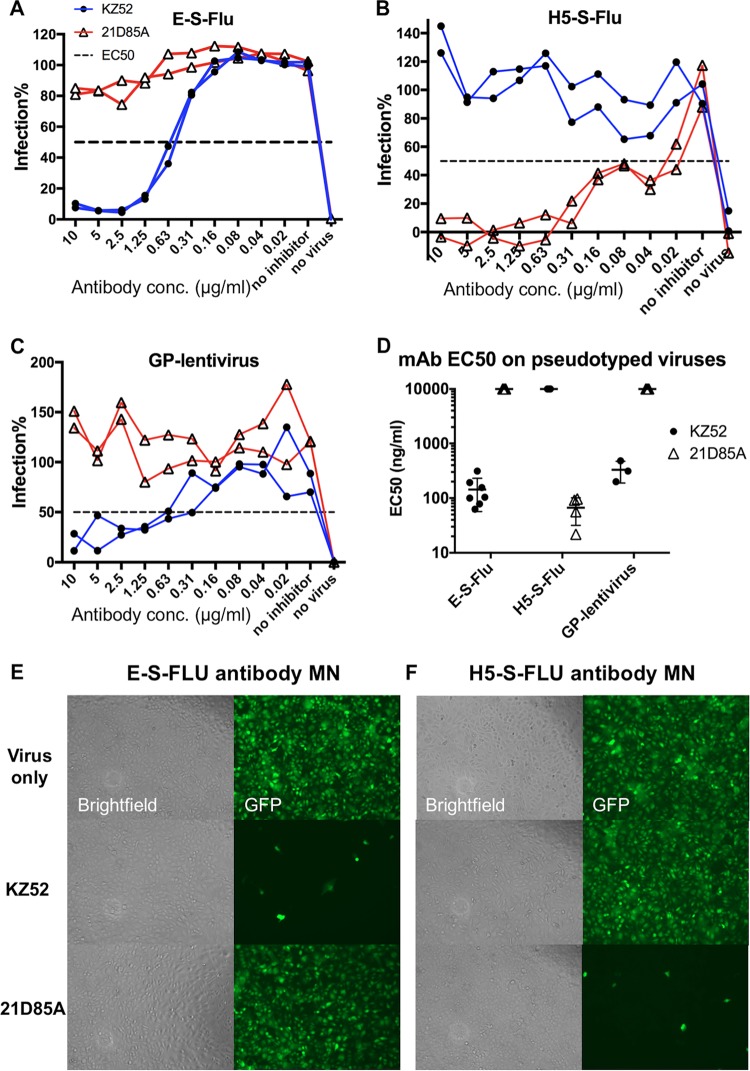
Antibody neutralization of pseudotyped E-S-FLU virus, H5-S-FLU virus, and EBOV-GP-pseudotyped lentivirus. Viruses were preincubated for 2 h with antibody KZ52 (anti-GP) or 21D85A (anti-H5) before MDCK-SIAT1 indicator cells were added. Antibodies were titrated in 2-fold dilutions from 10 μg/ml. After 24 h, infection was quantified by measuring eGFP expression (influenza virus) or luminescence (lentivirus). Titration curves are shown in panels A to C. A summary of the EC_50_s of MAbs KZ52 and 21D85A for the three pseudotyped viruses is shown in panel D. Multiple EC_50_s from repeat experiments are shown with the geometric means and 95% CIs. A value of 10,000 ng/ml was assigned to antibodies that did not neutralize. (E and F) Microscopy (10× objective) of assays showing the specificity of MN by antibodies. For “Virus only,” the E-S-FLU and H5-S-FLU viruses were added directly to MDCK-SIAT1 cells. Infected cells express eGFP, shown as green cells. In the MN assay, virus was preincubated with 10 μg/ml MAb KZ52 (anti-GP) or 21D85A (anti-H5) before the infection of MDCK-SIAT1 cells. Inhibition is visualized as a significantly reduced number of green fluorescent cells.

The neutralization profiles of two antibodies are shown in Fig. 4. KZ52 is a human MAb that specifically binds to the Zaire species of EBOV-GP and recognizes a conformational epitope at the base of the GP (35, 36). KZ52 neutralized the E-S-FLU virus and the GP-lenti pseudotyped virus with an EC_50_ similar to what has been reported for wild-type Ebola virus (300 ng/ml [35]), while it did not neutralize the H5-S-FLU virus. The neutralization effect was confirmed by fluorescence microscopy (Fig. 4E and F). In contrast, 21D85A, an H5-specific MAb, neutralized the H5-S-FLU virus but did not neutralize the E-S-FLU virus or the GP-lenti pseudotyped virus. This shows that the E-S-FLU virus-pseudotyped virus we produced is coated with correctly folded EBOV-GP and requires this molecule to infect the MDCK-SIAT1 indicator cells. These results show that the E-S-FLU virus can be used to screen for neutralizing antibodies to Ebola virus.

### Inhibition of E-S-FLU virus infection by small molecules.

In the literature, several drug screening studies have been done with wild-type Ebola virus (37–41) and Ebola virus surrogates such as Ebola virus-like particles (42), EBOV-GP-pseudotyped recombinant VSV (32), and lentivirus (43, 44). It was shown that drug molecules with diverse pharmacological functions have inhibited Ebola virus infection *in vitro*. To verify the use of the E-S-FLU virus in drug screening assays, we selected several types of drug molecules that have been tested repeatedly in the literature and screened them against the E-S-FLU virus in our drug inhibition assay. Table 1 summarizes the 13 initial inhibitors we tested. E-S-FLU virus infection was effectively blocked by different groups of drugs, and the drug concentrations at which eGFP fluorescence was reduced by 50% (IC_50_s) were in the same range as those in the literature.

**TABLE 1:** IC_50_s of 13 drugs that inhibited E-S-FLU virus infection selected from the literature

Drug (rank in Table S1)	Mechanism	Geometric mean IC_50_, μM^*a*^ (95% CI)	IC_50_ (μM) in literature	Virus	Reference	LogP	pKa^*b*^
Amiodarone (18)	Calcium channel inhibitor	2.04 (0.54∼7.76)	0.4	Wild-type EBOV	37	7.2	8.5
Verapamil (71)		10.6 (2.75∼9.8)	3.1	Wild-type EBOV	38	3.8	9.7
Tetrandrine (13)		1.63 (0.65∼4.07)	0.06	Wild-type EBOV	38	5.6	8.3
Clomiphene (6)	Estrogen receptor antagonist	0.35 (0.23∼0.55)	2.4	Wild-type EBOV	39	6.1	9.3
Toremifene (1)		0.15 (0.14∼0.16)	0.2	Wild-type EBOV	39	5.7	8.8
Imipramine (33)	Antidepressant	3.82 (2.51∼5.75)	13.7	EBOV (VLPs)	42	4.5	9.4
Clarithromycin (87)	Antibiotic	14.0 (2.19∼91.2)	4.5	EBOV (VLPs)	42	3.2	8.4
U18666A (21)	NPC1 phenotype inducer	2.32 (0.19∼28.8)	3.0	rVSV-EBOV-GP	32	5.5	9.4
Arbidol (39)	Fusion inhibitor	5.17 (0.60∼43.7)	5.97	Wild-type EBOV	40	5.1	9.9
EIPA (106)	Macropinocytosis inhibitor	17.8 (14.1∼22.4)	25.0	Wild-type EBOV	41	0.3	2.3
MDL28170 (32)	Calpain inhibitor	3.81 (0.85∼17.0)	10.0	GP-lenti	43	2.8	−4.3
E64 (201)	Cathepsin inhibitor	77.2 (64.6∼93.3))	50.0	GP-lenti	43	−1.1	11.3
Niclosamide (14)	Proton ionophore	1.69 (1.00∼2.88)	2.64	Wild-type EBOV	40	4.1	−4.4

a For E-S-FLU virus.

b Strongest basic.

**FIG 5:**
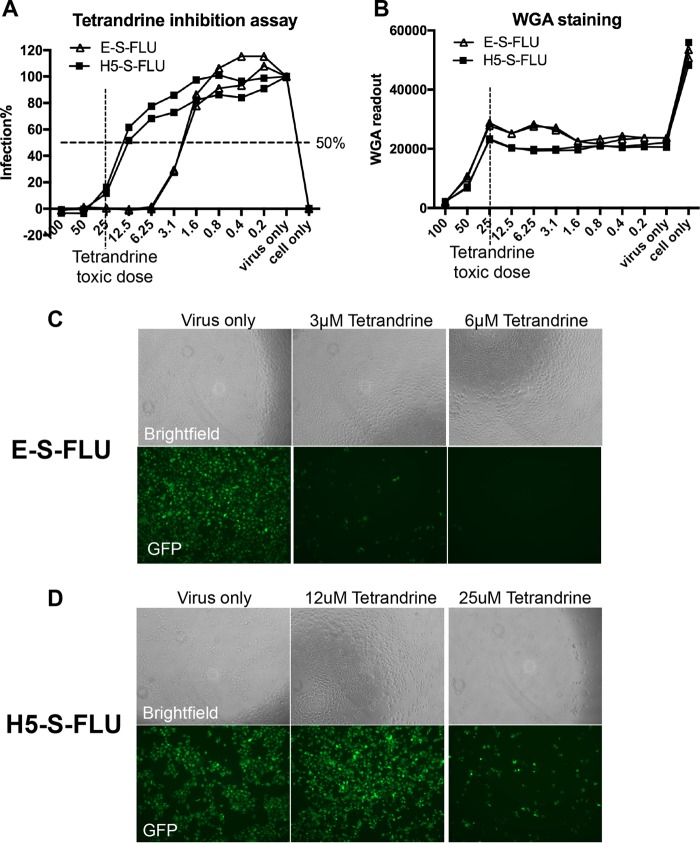
Tetrandrine inhibition assay with the E-S-FLU and H5-S-FLU viruses. Tetrandrine was titrated from 100 to 0.2 μM in the drug inhibition assay against the E-S-FLU and H5-S-FLU viruses. Cells were preincubated with tetrandrine for 3 h and infected with virus for 24 h. (A) Percent infection was quantified by eGFP expression measured with a fluorescence plate reader. (B) WGA was added to stain cell membrane sialic acid and *N*-acetylglucosaminyl residues as an estimation of the number of cells remaining in each well after fixation and washing. Toxicity reduces the WGA fluorescence as cells detach from the plastic. Note that the S-FLU virus expresses neuraminidase, which partially reduces WGA binding between “cell only” and “virus only” in panel B. Neutralization of the S-FLU virus is associated with some increase in WGA binding as neuraminidase expression is reduced. (C and D) Microscopy of tetrandrine inhibition assays. Note that the toxicity of tetrandrine at 25 μM resulted in a reduction in the number of cells in the bright-field channel.

All of the screenings in Table 1 were repeated at least three times to calculate the geometric mean IC_50_ and 95% confidence interval (CI). We also screened these drugs against the H5-S-FLU virus (see Table S1 in the supplemental material). Most of them did not inhibit the H5-S-FLU virus, except niclosamide at 2.64 μM. Figure 5 shows an example of drug inhibition by tetrandrine. The infection level was quantified by measuring eGFP expression. We also estimated the toxicity of each drug by staining the cells with fluorescently labeled wheat germ agglutinin (WGA) after infection, fixation, and washing. WGA is a lectin that binds to cell surface *N*-acetyl-d-glucosamine and sialic acid. Cells in good condition will remain adhered to the plate during analysis and can be detected with WGA. Dead or unhealthy cells will detach and be washed away. WGA staining allows quick indication of drug toxicity in high-throughput plate screening assays. This is verified by light microscopy (Fig. 5C and D).

The calculated logP and pKa values of these inhibitors are shown in Table 1. As found by others, many of the drug molecules that inhibited Ebola virus have high logP and pKa values (e.g., amiodarone, verapamil, and clomiphene) and can be classified as cationic amphiphilic drugs (37, 45). There are also noncationic amphiphiles that inhibited Ebola virus entry, such as 5-(*N*-ethyl-*N*-isopropyl)amiloride (EIPA) (41, 46), MDL28170 (43, 47), E64 (43, 44, 48–50), and niclosamide (40, 42). For the potential mode of action of each of these molecules, see Fig. 8.

### LOPAC^1280^ library screening.

Having established that infection with the E-S-FLU virus was dependent on the NPC1 receptor and was inhibited by specific antibodies and established small molecules, we proceeded to a formal drug screening. We screened 1,280 drug molecules from the LOPAC^1280^ library (Sigma-Aldrich) in our drug inhibition assay to test their effects on E-S-FLU and H5-S-FLU virus infections. This library contains 1,280 pharmacologically active small molecules with different molecular targets. All of the drugs were screened in the first round at 100 and 5 μM. Selected drugs were then titrated once in a drug inhibition assay to calculate their IC_50_s.

**FIG 6:**
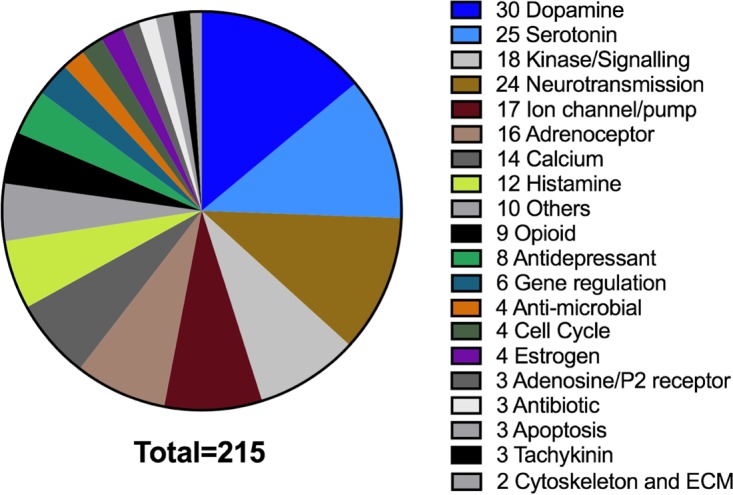
Groups of drugs from the LOPAC^1280^ library that inhibited the E-S-FLU virus. Of 1,280 small molecules, 215 inhibited E-S-FLU virus infection. They are grouped and displayed according to their pharmacological activities. The number of drug molecules in each group that inhibited the E-S-FLU virus is shown.

**FIG 7:**
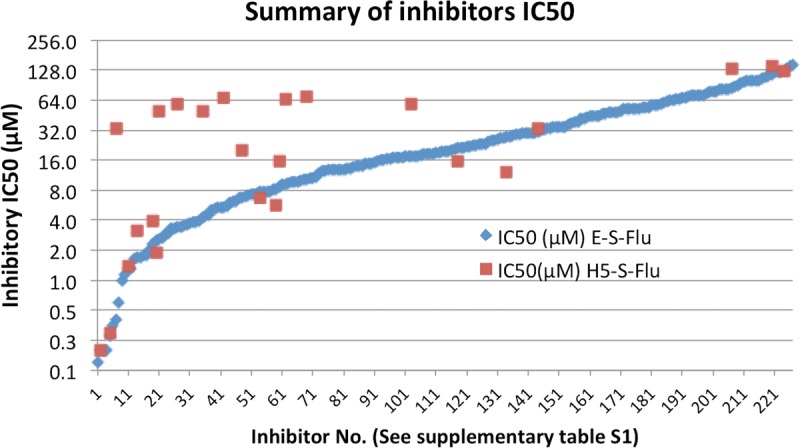
Summary of all of the drugs (228 molecules) that inhibited the E-S-FLU virus in this study. This includes 215 inhibitors from the LOPAC^1280^ library and 13 additional inhibitors tested. Twenty-five molecules inhibited the H5-S-FLU virus, all of which also inhibited the E-S-FLU virus. The inhibitors are ranked and plotted according to their IC_50_s (a titration example is shown in Fig. 5). For detailed information on the inhibitors, see Table S1.

In total, 215 out of 1,280 molecules inhibited E-S-FLU virus infection to some level before reaching toxicity *in vitro* (Fig. 6 and 7; see Table S1). Out of the 215 that inhibited the E-S-FLU virus, 58 had an IC_50_ of <10 μM. In comparison, only 22 of the 1,280 molecules inhibited the H5-S-FLU virus and 8 had IC_50_s of <10 μM. All of these 22 molecules (Table 2) also inhibited the E-S-FLU virus and thus were likely to be acting either on both EBOV-GP and influenza virus HA or at a postentry stage that is independent of the surface glycoprotein. We did not see any molecule that inhibited the H5-S-FLU virus but did not inhibit the E-S-FLU virus. A detailed profile of all the inhibitors is shown in Table S1. These 215 inhibitors belong to a variety of pharmacological groups according to the description on Sigma's website (Fig. 6). Table S1 also includes 13 small molecules tested in addition to the LOPAC^1280^ library, to give a total of 228 E-S-FLU virus inhibitors, and of these, 25 inhibited both the E-S-FLU and H5-S-FLU viruses (Fig. 7 and Table 2).

**TABLE 2:** Twenty-five small molecules^*a*^ that inhibited both the E-S-FLU and H5-S-FLU viruses in this study

No. in Table S1	LOPAC plate no.	Sigma catalog no.	Name	Highest concn (μM) tested	IC_50_ (μM) for:		Effect
					E-S-FLU	H5-S-FLU	
5	12-G4	O3125	Ouabain	1.5	0.28	0.3	Reduction
7	14-B2	R1402	Raloxifene HCl	50	0.40	33.8	Reduction
14	11-G7	N3510	Niclosamide	25	1.69	3.2	Reduction
21		U3633	U18666A	100	2.56	49.5	Reduction
27	04-B11	C8903	Clemastine fumarate salt	100	3.40	58.4	Reduction
35		SML0445	Clemastine	50	4.30	50.0	Reduction
42	04-F7	C6628	Chloroquine diphosphate	200	5.44	67.0	Reduction
48	03-B7	PZ0171	CP-100356 monohydrochloride	50	6.80	20.0	Reduction
54	02-H5	A9809	Amsacrine hydrochloride	25	7.68	6.76	Reduction
61	02-E10	A255	A-77636 hydrochloride	25	8.60	15.6	Reduction
63	08-E9	H127	Hexahydro-sila-difenidol hydrochloride, *p*-fluoro analog	100	9.30	65.0	Reduction
70	12-E7	L9793	LP44	100	10.40	70.0	Reduction
118	02-C7	PZ0178	PHA 767491 hydrochloride	25	21.30	15.7	Reduction
2	01-D5	A1784	Aminopterin	100	0.20	0.2	Dimmer
11	02-C11	SML0113	Brequinar sodium salt hydrate	100	1.30	1.4	Dimmer
19	04-D3	C3930	Calmidazolium chloride	6.25	2.30	3.9	Dimmer
20	03-D6	G6423	Gemcitabine hydrochloride	100	2.50	1.9	Dimmer
60	05-D6	D0670	Dihydroouabain	25	8.30	5.7	Dimmer
104	13-B10	SML0678	Pyridostatin trifluoroacetate salt	200	17.40	58.8	Dimmer
135	05-B9	D3768	Dequalinium chloride hydrate	100	27.30	12.1	Dimmer
145	14-C9	S3378	Spironolactone	100	30.40	33.1	Dimmer
159		2778-5^*b*^	Faviparivir (T705)	500	39.30	67.3	Dimmer
209	15-H7	T4143	Triamterene	200	85.20	132.3	Dimmer
222	15-A8	T4182	Tyrphostin AG 1478	200	115.30	141.0	Dimmer
226	14-C4	R9644	Ribavirin	200	125.20	125.9	Dimmer

a Twenty-two are from the LOPAC^1280^ library.

b Cambridge Biosciences.

In our screening system, we quantified infection by measuring the overall fluorescence level of cells. Under a fluorescence microscope, it was clear that most drugs reduced the number of fluorescent cells (i.e., reduced the proportion of infected cells) as the drug concentration increased. Twelve drugs, however, caused a reduced overall fluorescence level of infected cells without reducing the number of fluorescent cells significantly. All 12 of these drugs also affected H5-S-FLU virus infection. This suggests that they acted at a postentry phase by limiting the expression of viral proteins. In Table 2, they are annotated as “dimmer” drugs. The other 13 molecules that inhibited both the E-S-FLU and H5-S-FLU viruses reduced the number of infected cells and thus may have affected the function of both glycoproteins.

The IC_50_s of all 228 molecules that inhibited the E-S-FLU virus are plotted in Fig. 7. These include the 215 inhibitors from the LOPAC^1280^ library and the additional 13 individual drugs we tested in addition to this library.

### Comparison with the screening results of Johansen et al.

We compared our screening results with those recently reported by Johansen et al. (40), who used wild-type EBOV to screen for inhibitors. The two libraries have 538 drugs in common (Table 3). The results obtained with 442 of them agreed; 23 of these were inhibitors, and 419 had no effect. Of the rest of the drugs, 70 were positive in the LOPAC^1280^ screening only and 26 were positive in the screening done by Johansen et al. only. We used a two-tailed Fisher exact test for statistical analysis of the comparison, and the correlation was significant (*P* < 0.0001).

**TABLE 3:** Comparison of drugs common to both the LOPAC^1280^ library and the collection of Johansen et al.^*a*^

Set of drugs in LOPAC^1280^ library	No. of drugs in collection of Johansen et al.		
	Inhibitors	Noninhibitors	Total
Inhibitors	23	70	93
Noninhibitors	26	419	445
Both	49	489	538

a A total of 538 drugs appeared in both our LOPAC^1280^ library screening and that of Johansen et al. (40). Associations between the two screenings were calculated by two-tailed Fisher exact test (*P* < 0.0001).

One of the main differences between the two screenings is the range of drug concentrations tested. The highest concentration tested in the screening done by Johansen et al. was 13 μM, whereas in our screening, most of the drugs were screened at concentrations of >50 μM if toxicity was not reached. For this reason, the great majority of “agreements” occurred in the drugs with an IC_50_ of <10 μM in our assay (Table S1). For the drugs that were positive in the LOPAC^1280^ screening only, we selected ebastine (E9531), imipramine (I7379), and verapamil (V4629) and ordered them individually from Sigma to confirm their inhibitory effects. All three drugs had an inhibitory effect on the E-S-FLU virus with IC_50_s comparable to those in the LOPAC^1280^ library screening. All three drugs have previously been reported as inhibitors (32, 37, 38, 40).

Of the 26 drugs that were positive in the screening done by Johansen et al. but negative in our LOPAC^1280^ screening, we ordered individually from Sigma and further tested 14, auranofin (Sigma code A6733), bepridil (B5016), calcimycin (C7522), D609 (T8543), diphenyleneiodonium (D2926), doxylamine (D3775), linezolid (PZ0014), naproxen (M1275), nocodazole (M1404), perphenazine (P6402), rotenone (R8875), sanguinarine (S5890), serotonin (H9523), and thapsigargin (T9033).

Bepridil (a calcium channel inhibitor) and perphenazine (a cationic amphiphile) from the LOPAC^1280^ library did not inhibit the E-S-FLU virus, but the individually ordered samples showed specific inhibition of the E-S-FLU virus (bepridil IC_50_ = 2.7 μM, perphenazine IC_50_ = 8.2 μM). This indicates some variation due to false negatives in the library preparation from Sigma.

D609, doxylamine, disopyramide, linezolid, naproxen, rotenone, and sanguinarine were confirmed as noninhibitors of the E-S-FLU virus in our assay up to 100 μM. Auranofin, calcimycin, diphenyleneiodonium, nocodazole, and thapsigargin were toxic to MDCK-SIAT1 cells even at 0.2 μM, yet some cells were still infected. None of the drugs that were further tested inhibited the H5-S-FLU virus.

## DISCUSSION

The design of the E-S-FLU virus was based on a pseudotyped influenza virus, the S-FLU virus, that was previously developed by our group and is similar to other single-cycle influenza virus pseudotypes (12, 22, 51). The E-S-FLU virus is a nonreplicating pseudotyped virus and can be manipulated at biosafety level 1/2, as it does not contain any genetic information from Ebola virus. For ease of production, we found that the MDCK-SIAT1 cell line (21) can stably express full-length EBOV-GP without toxicity. Although replication of the S-FLU virus core in the EBOV-GP-transduced cell line is 2 to 3 orders of magnitude less efficient than that in H5 HA-expressing cells (Fig. 2), production of the pseudotype at a scale sufficient for high-throughput assays is simple and efficient. The E-S-FLU virus encodes eGFP as a reporter in preference to firefly luciferase or beta-galactosidase (9, 52, 53). Enzyme reporters are much more sensitive than eGFP, and very low-level infection can be amplified and detected. However, cells have to be lysed to release the enzymes and then substrate is added for a one-round readout only. In comparison, for drug inhibition and neutralization assays, the S-FLU virus can be added to indicator cells at a multiplicity of infection (MOI) of ≥1 to produce bright eGFP fluorescence in most cells that is stable over months after fixation and can be measured in a plate reader or observed by microscope without further manipulation (Fig. 4 and 5).

**FIG 8:**
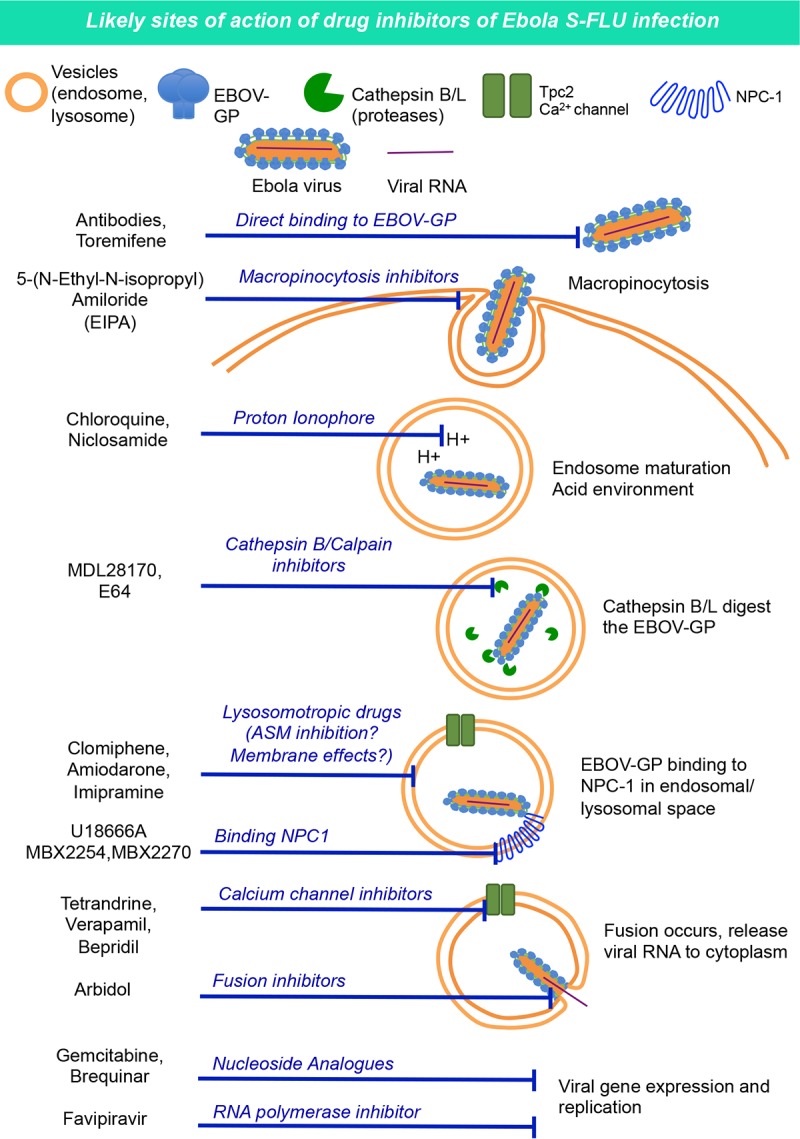
Ebola virus cell entry mechanism (94) and potential drug modes of action.

To validate the E-S-FLU virus as a useful surrogate for Ebola virus, we showed that infection is fully dependent on the NPC1 receptor (Fig. 3), is inhibited by the EBOV-GP-specific MAb KZ52 (35, 36) (Fig. 4), and is also inhibited by a well-characterized set of 13 drugs that are thought to act at various points during viral entry (37–42) (Table 1 and Fig. 8).

Several laboratories have observed that a wide variety of FDA-approved drugs can inhibit infection of cell lines *in vitro* by Ebola virus (40, 42). We have screened 1,280 drugs from the LOPAC^1280^ library to identify pharmacologically active small molecules that inhibit E-S-FLU virus cell entry. The H5-S-FLU virus was tested in parallel with the E-S-FLU virus to differentiate inhibition specific to EBOV-GP or to the S-FLU virus core. We identified 215 molecules from the library that inhibited the E-S-FLU virus, whereas only 22 inhibited the H5-S-FLU virus, all of which also inhibited the E-S-FLU virus. Therefore, the inhibitory effects of 193 drugs were EBOV-GP dependent.

We compared our screening results with the list of drugs screened by Johansen et al. (40), who screened 2,635 small molecules on GFP-expressing Ebola virus. A total of 538 small molecules were common to the two libraries (Johansen et al. versus LOPAC^1280^). Out of these, 442 molecules had concordant results in the two screenings. The correlation was statistically significant (*P* < 0.0001). It is noteworthy that we identified at least 17 calcium channel inhibitors as specific inhibitors of E-S-FLU virus infection. Independent evidence exists for a possible role for calcium channels in the lysosomal membrane in viral entry (37, 38), perhaps through an effect on vesicle fusion (32, 54, 55). Recent studies have suggested that two-pore channel 2 (TPC-2), a lysosomal calcium channel, is involved in the Ebola virus entry process, and this could be the target of the calcium channel inhibitors (38, 56).

A large group of inhibitors share a cationic amphiphilic feature, i.e., a hydrophilic end, usually a weak base amine group (high pKa) and a hydrophobic end (high logP), and are therefore likely to concentrate in lysosomes (57–59). The mechanism by which these drugs inhibit Ebola virus entry has not been resolved (37, 39). Some cationic amphiphilic drugs can cause an increase in cholesterol accumulation in endosomes and lysosomes, where EBOV-GP membrane fusion occurs. Others can influence lysosomal membrane stability and indirectly inhibit acid sphingomyelinase, which are also known as functional inhibitors of acid sphingomyelinase (60, 61), and may have an indirect effect on calcium mobilization from the lysosome through the accumulation of sphingosine. In addition, the most powerful member of the cationic amphiphilic group, toremifene, has been shown to bind to a hydrophobic pocket of the EBOV-GP and destabilize the molecule *in vitro* (62) and so may have two sites of action.

Many of these drugs have been found to have a risk of causing long-QT syndrome, such as amiodarone, toremifene, and clarithromycin (63, 64). Inhibition of the potassium channel hERG (human ether-a-go-go) in cardiac myocytes is commonly associated with long-QT syndrome (65). By comparing the 228 drugs that inhibited the E-S-FLU virus in our studies with the hERG screening literature (65–67), we identified at least 46 (20.2%; indicated in Table S1) as confirmed inhibitors of the hERG channel and hence may have this dangerous side effect on cardiac conduction. Most of these hERG binders are typical cationic amphiphilic drugs. It is unclear whether the features that inhibit Ebola virus entry also predispose to the effect on hERG binding and lengthening of the QT interval. Further dissection of their mechanisms of action is therefore necessary before they can be administered to Ebola virus-infected patients, although they are FDA-approved drugs (68, 69). As more potential mechanisms of action of these drugs are uncovered, testing of combinations that act at different sites for synergistic effects may be valuable to reduce this risk (70, 71).

One advantage of our S-FLU virus pseudotype is that infected cells express eGFP and fluoresce brightly. Visual inspection of cells in assay plates by fluorescence microscopy revealed that two forms of inhibition could be distinguished. In most cases, the drugs reduced the number of infected cells. All of the 203 drugs that acted in an EBOV-GP-dependent manner reduced the number of infected cells, which is consistent with inhibition of viral entry (Table S1). In contrast, in some cases, the drugs did not reduce the number of infected cells but reduced the intensity of fluorescence expression in the infected cell population. Of the 228 drugs tested, 12 reduced the fluorescence intensity (Table 2). All of these also inhibited the H5-S-FLU virus. In general, the IC_50_s of these agents were similar for the E-S-FLU and H5-S-FLU viruses, which is consistent with their acting on functions controlled by the influenza virus core, which is common to both. We suggest that they affect the virus at a postentry level like faviparivir, a specific inhibitor of RNA-dependent RNA polymerase (72–75) that was initially synthesized to inhibit influenza virus but later found to be effective against Ebola virus *in vitro*. In addition aminopterin, a dihydrofolate reductase inhibitor; brequinar, a dihydroorotate dehydrogenase inhibitor (76–79); and ribavirin, a guanosine analogue nucleoside inhibitor (71, 80, 81), may affect transcription indirectly by blocking the biosynthesis of nucleotides. It is interesting that spiranalactone and triamterene both have the dimmer effect. These drugs are used clinically as diuretics that block sodium channels. It is unclear how they can affect the functions of the influenza virus core at a postentry level.

Of the 25 drugs that inhibited both the H5-S-FLU and E-S-FLU viruses, 13 reduced the number of infected cells and thus may have inhibited cell entry by both H5 HA and EBOV-GP. Of these, chloroquine (a lysosomotropic drug) and niclosamide may act by reducing the lysosomal proton concentration, which can influence the efficiency of cathepsin in endosome-digesting EBOV-GP (82). Niclosamide is a proton ionophore (83, 84) and had similar IC_50_s on H5-S-FLU virus (3.3 μM) and E-S-FLU virus (1.7 μM) entry. Arbidol may inhibit the fusion of viral and lysosome membranes in a manner related to its effect on influenza virus HA (85–87). Although we did not find inhibition by arbidol of the H5-coated S-FLU virus, we have since confirmed the inhibition of S-FLU virus coated with H7 HAs from A/Netherlands/219/2003 and A/Shanghai/1/2013/H7, as well as H1 HAs from A/PR/8/1934 and A/England/195/2009 (data not shown). A recent structure revealed arbidol bound to H7 HA from influenza virus (88); it may be interesting to analyze the binding of arbidol to EBOV-GP.

In summary, we have developed a new surrogate E-S-FLU virus for Ebola virus. It mimics the Ebola virus at the level of cell entry and can be a valuable tool for the screening of antibodies and therapeutic drugs, as well as studying the fundamental biology of the Ebola virus entry process.

## MATERIALS AND METHODS

### Pseudotyped virus design and production.

cDNA encoding EBOV-GP from Zaire ebolavirus Makona wt/GIN/2014/Kissidougou-C15 (GenBank accession no. KJ660346.1) was human codon optimized and synthesized by GeneArt. Lentiviral vector pHR-SIN (89) was engineered to incorporate the EBOV-GP cDNA. We transduced Madin-Darby canine kidney (MDCK-SIAT1) epithelial cells (21) with lentivirus to express full-length EBOV-GP.

Transduced cells were stained with EBOV-GP-specific MAb KZ52, which is specific to Zaire (35, 36), or 66-4-C12, which is cross-reactive with all Ebola virus species (P. Rijal, unpublished data), and with a second layer of fluorescein isothiocyanate (FITC)-conjugated goat anti-human antibody (Life Technologies reference no. H10301). Stained cells were bulk sorted with a fluorescence-activated cell sorter (FACS) to achieve maximal expression of EBOV-GP (E-SIAT cell line, shown in Fig. 1). EBOV-GP was detected on >99% of the sorted cells and was stable and uniform up to at least to the ninth passage after sorting.

The E-S-FLU virus was generated on the basis of a nonreplicating pseudotyped influenza virus (S-FLU) that was previously described (12, 90). We used the version that expresses enhanced green fluorescent protein (S-eGFP), which has its S-HA coding sequence replaced with an eGFP reporter gene. We isolated subclones of the original S-FLU virus that showed brighter fluorescence and found a single T60C mutation in the A/PR/8/34 HA signal sequence (NCBI reference sequence accession no. NC_002017.1) that abrogates an out-of-frame ATG, which had interfered with downstream eGFP expression. All subsequent versions of the S-FLU virus contain this enhancement, which gives a sufficiently bright fluorescence signal from infected cells to read on the CLARIOstar fluorescence plate reader. Seed viruses were doubly cloned by limiting dilution before the seeding of E-SIAT or H5-SIAT producer cells.

Seed S-FLU viruses coated in H5, H7, or H1 HA were titrated by standard 50% tissue culture infective dose (TCID_50_) assay on MDCK-SIAT1 cells by fluorescence detection (as described below). Titration experiments revealed that a relatively high MOI of approximately 1 TCID_50_ of seed S-FLU virus per cell added to infect E-SIAT cells produced the highest titers of the E-S-FLU virus, whereas a lower MOI (∼0.01) was adequate for seeding of HA-SIAT cell lines (12). The viral growth medium (VGM) used was Dulbecco's modified Eagle's medium (DMEM) with 0.1% bovine serum albumin, 10 mM HEPES, 2 mM glutamine, 100 U/ml penicillin, and 100 μg/ml streptomycin. After 2 h of incubation of E-SIAT cells with seed S-FLU virus, any remaining seed virus was removed by washing with phosphate-buffered saline (PBS). Cells were then incubated in VGM for 48 h at 37°C in a 5% CO_2_ incubator without added trypsin. Culture supernatant was harvested and stored in aliquots at −80°C. Replication of HA-pseudotyped viruses required the addition of trypsin as described previously (12).

### Seed virus TCID_50_.

Seed S-FLU viruses coated in influenza virus HA were titrated for the concentration of replication-competent clones (TCID_50_) by a standard limiting-dilution method as described by Powell et al. (12). Wells containing clones of eGFP-expressing S-FLU virus >4 standard deviations above 16 control wells were detected with a plate reader (described below), and the TCID_50_/ml was calculated by the Reed-Muench method (91).

### S-FLU virus batch CID_50_.

Batches of the E-S-FLU and H5-S-FLU viruses were titrated for infectivity of MDCK-SIAT1 indicator cells after overnight infection. Harvested culture supernatants (100 μl) containing the E-S-FLU or H5 S-FLU virus were titrated in 2-fold dilutions in VGM across a flat-bottom 96-well plate (Fig. 2A). MDCK-SIAT1 indicator cells were washed in PBS, and 3 × 10^4^ cells in 100 μl of VGM were added to each well and incubated for 18 h (37°C, 5% CO_2_). Medium was removed from the wells, and cells were fixed in 10% formalin (in PBS) for 30 min at 4°C. The virus titer was assessed by eGFP fluorescence analyzed with a plate reader (CLARIOstar). The dilution of virus giving 50% of the maximum plateau fluorescence signal (EC_50_) was calculated by linear interpolation. The EC_50_ dilution obtained by best fit of the titration curve to the Poisson distribution was very similar (data not shown). The typical EC_50_ dilutions for the E-S-FLU virus harvested after 48 h were ∼1:8, compared to ∼1:500 for the H5-S-FLU virus (Fig. 2). From the EC_50_ dilution and the number of cells per well (3 × 10^4^), the CID_50_/ml was calculated as ∼2 × 10^6^ in the E-S-FLU virus batch and ∼1.6 × 10^8^ in the H5 S-FLU virus batch. For inhibition assays, the H5-S-FLU and E-S-FLU viruses were used at a concentration that gave close-to-maximum plateau fluorescence in infected MDCK-SIAT1 cells (MOI, ∼4 CID_50_/cell). This value was used to normalize the fluorescence readout to a percentage of the maximum in the figures (Fig. 4A and B and 5A).

In this study, all of the evaluation and screening was done with the Zaire (C15) E-S-FLU and H5-S-FLU viruses in parallel.

### CLARIOstar plate reader setting.

Fluorescence plate readout was performed with Costar 96-well cell culture plates (Costar no. 3799). We chose the COSTAR 96 microplate mode in the CLARIOstar software. For measurement of eGFP levels, fluorescence excitation was at 483 nm (bandwidth of 8 nm) and emission was at 515 nm (bandwidth of 8 nm) with a 499-nm dichroic filter. Each well had 50 flashes at a 4-mm diameter and fluorescence readout from the top optic, giving the orbital averaging value. For measurement of WGA (Alexa Fluor 647 conjugate) in the drug inhibition assay, fluorescence excitation was at 630 nm (bandwidth of 30 nm) and emission was at 678 nm (bandwidth of 20 nm). Each well had 53 flashes at 4 mm and fluorescence readout from the top optic, giving the orbital averaging value. The focal length and gain were adjusted before each plate was read. The generally optimal focal length was 5.0 mm, the gain of the eGFP channel was adjusted to 3,000, and the gain for WGA-647 was adjusted to 2,500.

### E-S-FLU virus infection of NPC1 KO cells.

The NPC1 protein is the key receptor for Ebola virus cell entry at the level of the lysosomal membrane (31–33). Two NPC1 KO HeLa cell lines (ex2 NPC1-KO and ex4 NPC1-KO, named for the exon that was deleted) were generated by the CRISPR-cas9 technique. Generation and verification of the NPC1 KO cell lines have been described previously by Tharkeshwar et al. (34).

A total of 1 × 10^5^ wild-type, ex2 NPC1-KO, or ex4 NPC1-KO HeLa cells were seeded into a 24-well tissue culture plate in 500 μl of D10 medium (DMEM with 10% fetal calf serum, 2 mM glutamine, 100 U/ml penicillin, and 100 μg/ml streptomycin) the night before infection. Before infection, the medium was removed and cells were washed once with PBS. Cells were infected with maximal doses of 1 × 10^6^ CID_50_ of the E-S-FLU virus (MOI, ∼10 CID_50_/cell) or 8 × 10^7^ CID_50_ of the H5-S-FLU virus (MOI, ∼800 CID_50_/cell) and incubated overnight. After infection, cells were trypsinized and transferred to 5-ml polypropylene tubes. Percent infection was analyzed by counting fluorescent cells in a flow cytometer (CyAn ADP analyzer).

### MN assay.

The MN assay used measures the inhibition titer of antibodies against virus entry *in vitro*. The assay was done as described by Powell et al. (12, 92), with minor modifications. The E-S-FLU (MOI, ∼4 CID_50_/cell) and H5-S-FLU (MOI, ∼4 CID_50_/cell) viruses were diluted in VGM to give plateau expression of eGFP in 3 × 10^4^ MDCK-SIAT1 cells after overnight infection in 96-well flat-bottom plates. A 50-μl volume of virus was preincubated with 50 μl of antibody at 2-fold dilutions for 2 h. A total of 3 × 10^4^ MDCK-SIAT1 cells in 100 μl of VGM were then seeded into each well, and overnight infection was allowed (37°C, 5% CO_2_). After infection, the medium was removed from the wells and the cells were fixed with 100 μl of 10% formalin (in PBS) for 30 min at 4°C; the formalin was then replaced with 100 μl of PBS. The plates were analyzed with a fluorescence plate reader (CLARIOstar) and by fluorescence microscopy. The IC_50_ was calculated by linear interpolation. KZ52 is a human MAb that specifically binds Zaire EBOV-GP and neutralizes wild-type EBOV *in vitro* (35, 36, 93). Our version was reconstituted from the sequence published in the crystal structure PDB file (PDB code 3CSY) (36). 21D85A is a human MAb made in-house that specifically binds to H5 HA and neutralizes the H5-S-FLU virus.

The EBOV-GP-pseudotyped lentivirus (GP-lenti) we used in this study is coated with the same EBOV-GP from Zaire C15 as the E-S-FLU virus. GP-lenti carries a firefly luciferase reporter gene. Fifty-microliter volumes of GP-lenti pseudotyped virus were preincubated with 50 μl of antibodies at 2-fold dilutions for 2 h, and 3 × 10^4^ MDCK-SIAT1 cells were added. Cells were incubated with virus for infection in VGM for 48 h. After 48 h, infection was quantified by measuring luciferase reporter expression. Medium was removed from the wells, and cells were lysed with 50 μl of Glo-lysis buffer (Promega) for 5 min, and then 50 μl of Bright-Glo (Promega) substrate was added for a 5-min enzymatic reaction. Solutions were transferred to opaque plates for luminescence measurement with the GloMax-Multi detection system (Promega).

### Drug inhibition assay.

All small inhibitor molecules, including the LOPAC^1280^ library used in this study, were ordered from Sigma-Aldrich and stored in 10 mM dimethyl sulfoxide at −20°C, with the exception of AY9944, which was ordered from Merck (catalog no. 190080), and trans-Ned19 (provided by our collaborator Antony Galione). Drugs were diluted in VGM to give a starting concentration of 100 μM (unless stated otherwise) and then titrated in 50 μl of VGM at 2-fold dilutions across a 96-well plate. A total of 3 × 10^4^ MDCK-SIAT1 cells were seeded in 50 μl of VGM and preincubated with drugs for 3 h (37°C, 5% CO_2_). A 100-μl volume of the E-S-FLU (MOI, ∼4 CID_50_/cell) or H5-S-FLU (MOI, ∼4 CID_50_/cell) virus diluted in VGM to give plateau expression of eGFP in 3 × 10^4^ MDCK-SIAT1 cells was then added for overnight infection. Cells were fixed with 100 μl of 10% formalin (in PBS) for 30 min at 4°C, the formalin was then replaced with 100 μl of PBS, and the plates were analyzed with a fluorescence plate reader (CLARIOstar) as for the MN assay. Infection and inhibition were quantified by measurement of the eGFP expression level. When more than three measurements were made for a drug, the 95% CI was calculated (Table 1).

Because of the toxic effect on cells of many drug molecules at high concentrations, dead cells were washed away before plate reading. This may give a falsely low eGFP fluorescence reading in the plate reader but can be detected by visual inspection in a fluorescence microscope. To estimate the number of cells remaining in each well, plates were stained with WGA after cells were fixed and washed once with PBS. WGA is a lectin that binds to sialic acid and *N*-acetylglucosaminyl residues at the cell surface. A 50-μl volume of 5 μg/ml WGA (Alexa Fluor 647 conjugate; ThermoFisher catalog no. W32466) was added to each well and incubated at room temperature for 15 min. Plates were then washed twice with PBS before being read (see the plate reader setting mentioned above).

### LOPAC^1280^ versus Johansen et al. drug screenings.

Johansen et al. (40) screened 2,635 compounds for antiviral activity against a genetically engineered Ebola virus that expresses eGFP (eGFP-EBOV) *in vitro*. In their Table S1, they listed all of the experimental data for the small molecules they screened in their study. Of the 2,635 molecules they tested, 538 were also found in the sigma LOPAC^1280^ library and were screened against the E-S-FLU virus in our study. We compared the inhibitory effects of these 538 compounds in both screenings (i.e., each molecule was either positive [inhibited infection] or negative [did not inhibit infection in the concentration range tested]) and performed a Fisher exact test to measure the significance of the association.

### LogP and pKa calculations.

For the drug molecules listed in Table 1, logP (partition parameter) and pKa values (strongest basic pKa) were estimated on the basis of their chemical structures by using the MarvinSketch program.

## Supplementary Material

Supplemental material
