# Supplementary material for: Characterization of Influenza Virus Pseudotyped with Ebolavirus Glycoprotein
Source: J Virol. 2018 Jan 30;92(4):e00941-17. doi: 10.1128/JVI.00941-17 (PMC5790926; doi:10.1128/JVI.00941-17)
Supplement: Supplemental material [file supp_92_4_e00941-17__index.html]

Supplemental material 

# Characterization of Influenza Virus Pseudotyped with Ebolavirus Glycoprotein

## Supplemental material

- Supplemental file 1 -

  Table S1 (Detailed information on all 228 drugs screened in this study.)

  XLSX, 176K
